# Vaginal microbiota evaluation and prevalence of key pathogens in ecuadorian women: an epidemiologic analysis

**DOI:** 10.1038/s41598-020-74655-z

**Published:** 2020-10-27

**Authors:** Ana María Salinas, Verónica Gabriela Osorio, David Pacha-Herrera, Juan S. Vivanco, Ana Francisca Trueba, António Machado

**Affiliations:** 1 Instituto de Microbiología, Colegio de Ciencias Biológicas y Ambientales (COCIBA), Universidad San Francisco de Quito (USFQ), Diego de Robles y Vía Interoceánica, Campus Cumbayá, Casilla Postal 17-1200-841, Quito, 170901 Ecuador; 2 Brain and Behavior Laboratory, Colegio de Ciencias de la Salud, Universidad San Francisco de Quito (USFQ), Diego de Robles y Vía Interoceánica, Campus Cumbayá, Casilla Postal 17-1200-841, Quito, 170901 Ecuador

**Keywords:** Clinical microbiology, Microbial communities, Microbiome

## Abstract

Vaginal infection is a gynecological problem in women of reproductive age with multiple health outcomes. The most common forms of infection include bacterial vaginosis (BV), vulvovaginal candidiasis (VC), and aerobic vaginitis (AV). Our main goals were to evaluate different types of vaginal infections in Ecuadorian women in a large urban area (Quito) and to characterize the vaginal microbiota colonization by opportunistic species. We collected vaginal swabs and epidemiological surveys from 414 women from June 2016 to July of 2017. We analyzed vaginal samples for the presence of any vaginal infection. The microbiological examination was done through Gram-stain, wet mount smears, and polymerase chain reaction (PCR) assays using primers for target genes, such as *16S* rRNA (*Atopobium vaginae, Mobiluncus mulieris,* and *Gardnerella* species), *ddl* (*Enterococcus faecalis*), *adk* (*Escherichia coli*) and *KER1* (*Candida albicans*) genes. Most women showed a healthy vaginal microbiota (66.7%). Nearly one-tenth (10.4%) of the participants had intermediate microbiota, and the remaining women (22.9%) had a single vaginal infection (BV, AV, or VC) or coinfections. From the 95 participants that had an infection, AV was the main diagnosed vaginal infection (51.6%), followed by BV (24.2%) and finally VC (7.4%). The remaining women (16.8%) showed coinfections, being BV and AV the most common coinfection. Using univariable logistic regression analyses we found an increased odds of healthy microbiota in women with a sexual partner (*P* = 0.02, OR = 1.64). Also, women in a free union relationship (*P* = 0.000, OR = 16.65) had an increased odds of having coinfections. On the other hand, the use of birth control (condom OR = 0.388 or other contraceptive method OR = 0.363) was associated with significantly lower odds of intermediate microbiota (*P* ≤ 0.05). We found no statistically significant differences between women with infection and a particular group age. Using multivariate logistic regression analyses we initially found an increased odds of having BV in women with *M. mulieris* (*P* = 0.020, OR = 4.98) and *Gardnerella* species (*P* = 0.010, OR = 4.16). Women with *E. coli* showed an increased odds of having AV (*P* = 0.009, OR = 2.81). The presence of *C. albicans* in women showed an increased odds of having VC (*P* = 0.007, OR = 17.94). Finally, women with *M. mulieris* showed a reverse odds of having healthy microbiota (*P* = 0.008, OR = 0.06). We found no statistically significant differences between women with symptomatic and asymptomatic infections or the presence of *Enterococcus faecalis*. We found using logistic regression analyses that *M. mulieris* was the most prevalent opportunistic pathogen among women with vaginal infection. Further studies should evaluate the possibility to use *M. mulieris* as a potential key predictor for vaginal infections.

## Introduction

Pregnancy outcomes and women’s health usually depend on the type of vaginal microbiota^1,2^. This microbiota consists of a dynamic ecosystem of various microbes in different quantities and ratios, can either protect the vaginal epithelium or cause different vaginal infections^3^. When there is a *Lactobacillus*-dominated vaginal microbiota, multiple *Lactobacillus* species reside in the vaginal epithelium as commensal bacteria and may act as the first defense mechanism against infection^4,5^. These lactobacilli act as biological surfactants preventing the initial adhesion of potential pathogens^6,7^. However, little is known about other types of vaginal microbiota in which *Lactobacillus* species do not dominate.

When this ecosystem gets disrupted, the vaginal epithelium is less protected, and vaginal infection sets in. Typically, vaginal infections are characterized by a shift in microbial communities that include a progressive replacement of certain *Lactobacillus* species by pathogenic or opportunistic microorganisms^3,4^. This microbial shift can lead to different vaginal infections such as bacterial vaginosis (BV) usually associated with several anaerobic or facultative bacteria, the most prevalent being: *G. vaginalis*; *Atopobium* sp.; *Prevotella* sp.; *Bacteroides* sp.; *Peptostreptococcus* sp.; *Mobiluncus* sp.; *Sneathia* sp.; *Leptotrichia* sp.; and genital *Mycoplasma*, such as *Mycoplasma hominis* and *Ureaplasma urealyticum*^4,8^. Another vaginal infection is vulvovaginal candidiasis (VC) due to *Candida albicans*, *Candida glabrata,* and *Candida tropicalis*^9^. Finally, aerobic vaginitis (AV) can be frequently caused by *E. coli*, *E. faecalis*, among other aerobic bacteria^3^.

Vaginal infection is considered the most prevalent gynecological problem of women of reproductive age, affecting millions every year, and the most common cause of gynecological medical care^10^. BV is associated with numerous health problems such as pelvic inflammatory disease, cervicitis, preterm labor, low birth weight, miscarriages, and chorioamnionitis^8,11–14^. Also, AV and BV are often associated with an increased risk of acquiring human immunodeficiency virus (HIV), Herpes simplex type 2, and other sexually transmitted infections with *Chlamydia trachomatis*, *Neisseria gonorrhoeae*, *Trichomonas vaginalis*, among others^12,15^.

Previous studies reported BV as the leading cause of vaginal infection in symptomatic women (22–50%), followed by VC (17–19%) and finally AV (approximately 11%)^4,9,16,17^. A variety of different risk factors, such as ethnicity and geographic location, have been found to influence the prevalence of BV. Several authors reported different BV prevalence in Asia, Europe, Africa, and Latin America^4,18–20^. However, in Ecuador, little is known about the prevalence of BV and other vaginal infections among women^20–22^.

The classical and clinical gold standard methods for vaginal infection diagnosis are physical examination, self-reported symptoms, pH of vaginal fluid, microscopy, and the whiff test^23–25^, which are usually applied in hospitals and clinical facilities worldwide^9^. Meanwhile, the gold standard in the (research) laboratory for the diagnosis of bacterial vaginosis is the Nugent score^20,26,27^. Although these techniques are highly sensitive and specific for evaluating BV in women^16^, they are not sensitive to characterize the composition of the vaginal microbiota. To avoid these drawbacks of the classical techniques, molecular analyses have been applied in multiple studies to better understand and characterize the microbiota present in healthy vaginal epithelium and vaginal infection^6,11,28–30^.

In this study, we applied classical and molecular microbiological techniques for the diagnosis of different types of vaginal infection, including microscopy and PCR assays^11,16,31^. We analyzed the prevalence of BV, VC, and AV in Ecuadorian women of reproductive age around the Quito area. Also, the present study aimed to elucidate the prevalence of symptomatic and asymptomatic vaginal infections in the study population and finally to characterize the vaginal microbiota colonization by several opportunistic species (*Atopobium vaginae, Mobiluncus mulieris*, *Gardnerella* species, *Enterococcus faecalis*, *Escherichia coli,* and *Candida albicans*).

## Results

### Epidemiological characteristics

A total of 414 women participated in this study. The exclusion criteria for the study included the absence of a legible and full disclosure survey or an inadequate result in DNA quantification [DNA concentration lower than 20 ng/µl; ratios of absorbance lower than 1.8 of phenolic compounds or the presence of salts (260/230 nm) and protein contaminants (260/280 nm)] for PCR assays (see Supplementary Table 1), and Gram staining procedures. Four hundred and fourteen women delivered the medical survey and a vulvovaginal swab sample for the diagnosis of vaginal infections (see Supplementary Table 2). The women were between 18 and 56 years old, and most of them were 21–30 years old (61.8%) (see Table 1). The majority were women of mestizo ethnicity (96.9%). Approximately 79.7% of study participants had a secondary (high school) level of education, being most of them undergraduate students (74.2%) or professionals (17.9%). The categories of professionals included: health professionals, administrative clerks, education, and general employees with college degrees. The majority of volunteers were single women (82.9%) and followed by married women (12.8%). Among the participants of our study set, 59.4% of the women reported having a sexual partner.

**Table 1:** Sociodemographic, behavioral variables among women in this study with healthy microbiota, intermediate microbiota, bacterial vaginosis, aerobic vaginitis, candidiasis, and coinfections.

	Healthy microbiota *N* (%)	Intermediate microbiota *N* (%)	Bacterial vaginosis *N* (%)	Aerobic vaginitis *N* (%)	Candidiasis *N* (%)	Coinfections *N* (%)	Total *N* (%)
Total incidence	276 (66.7)	43 (10.4)	23 (5.6)	49 (11.8)	7 (1.7)	16 (3.9)	414 (100.0)
**Age**							
Under 20	57 (20.7)	9 (20.9)	5 (21.7)	14 (28.6)	2 (28.6)	2 (12.5)	89 (21.5)
21–30	175 (63.4)	27 (62.8)	14 (60.9)	26 (53.1)	5 (71.4)	9 (56.3)	256 (61.8)
31–40	27 (9.8)	3 (7.0)	3 (13.0)	3 (6.1)	0 (0.0)	4 (25.0)	40 (9.7)
41–50	13 (4.7)	2 (4.7)	1 (4.3)	1 (2.0)	0 (0.0)	1 (6.3)	18 (4.3)
Over 50	4 (1.4)	2 (4.7)	0 (0.0)	5 (10.2)	0 (0.0)	0 (0.0)	11 (2.7)
**Ethnicity**							
Mestizo	269 (97.5)	42 (97.7)	22 (95.7)	48 (98.0)	6 (85.7)	14 (87.5)	401 (96.9)
Caucasian	3 (1.1)	0 (0.0)	0 (0.0)	0 (0.0)	1 (14.3)	2 (12.5)	6 (1.4)
Indigenous	4 (1.4)	1 (2.3)	0 (0.0)	1 (2.0)	0 (0.0)	0 (0.0)	6 (1.4)
Afro-Ecuadorian	0 (0.0)	0 (0.0)	1 (4.3)	0 (0.0)	0 (0.0)	0 (0.0)	1 (0.2)
**Education level**							
≤ Basic	4 (1.4)	1 (2.3)	0 (0.0)	1 (2.0)	0 (0.0)	0 (0.0)	6 (1.4)
Secondary	224 (81.2)	32 (74.4)	19 (82.6)	39 (79.6)	6 (85.7)	10 (62.5)	330 (79.7)
≥ University	48 (17.4)	10 (23.3)	4 (17.4)	9 (18.4)	1 (14.3)	6 (37.5)	78 (18.8)
**Occupation**							
Housewife	4 (1.4)	1 (2.3)	0 (0.0)	2 (4.1)	0 (0.0)	0 (0.0)	7 (1.7)
Student	212 (76.8)	30 (69.8)	19 (82.6)	33 (67.3)	5 (71.4)	8 (50.0)	307 (74.2)
Unprofessional	12 (4.3)	4 (9.3)	1 (4.3)	5 (10.2)	1 (14.3)	3 (18.8)	26 (6.3)
Professional	48 (17.4)	8 (18.6)	3 (13.0)	9 (18.4)	1 (14.3)	5 (31.3)	74 (17.9)
**Civil status**							
Single	229 (83.0)	36 (83.7)	20 (87.0)	41 (83.7)	7 (100.0)	10 (62.5)	343 (82.9)
Free Union (couples living together for at least 3 years without being married)	4 (1.4)	1 (2.3)	0 (0.0)	1 (2.0)	0 (0.0)	3 (18.8)	9 (2.2)
Married	39 (14.1)	5 (11.6)	2 (8.7)	5 (10.2)	0 (0.0)	2 (12.5)	53 (12.8)
Divorced	4 (1.4)	1 (2.3)	1 (4.3)	2 (4.1)	0 (0.0)	1 (6.3)	9 (2.2)
**Sexual partner**							
Not having	101 (36.6)	25 (58.1)	10 (43.5)	20 (40.8)	5 (71.4)	7 (43.8)	168 (40.6)
Having	175 (63.4)	18 (41.9)	13 (58.5)	29 (59.2)	2 (28.6)	9 (56.3)	246 (59.4)
**Contraceptive use**							
No	101 (36.6)	26 (60.5)	7 (30.4)	19 (38.8)	2 (28.6)	7 (43.8)	162 (39.1)
Yes	175 (63.4)	17 (39.5)	16 (69.6)	30 (61.2)	5 (71.4)	9 (56.3)	252 (60.9)
**Birth control methods**							
Condom	82 (29.7)	7 (16.3)	11 (47.8)	17 (34.7)	4 (57.1)	4 (25.0)	125 (30.2)
Hormonal contraception	47 (17.0)	2 (4.7)	2 (8.7)	6 (12.2)	1 (14.3)	3 (18.8)	61 (14.7)
Combined	38 (13.8)	6 (14.0)	2 (8.7)	5 (10.2)	0 (0.0)	2 (12.5)	53 (12.8)
Others	8 (2.9)	2 (4.7)	1 (4.3)	2 (4.1)	0 (0.0)	0 (0.0)	13 (3.1)
None or don’t answer	101 (36.6)	26 (60.5)	7 (30.4)	19 (38.8)	2 (28.6)	7 (43.8)	162 (39.1)

*N* number of women who responded in the survey within each category; *%* assigned percentage for each classification within each category.

Finally, concerning birth control methods, 39.1% of the women declared no use of contraceptive method or did not answer this question. While 30.2% and 14.7% of participants reported to strictly use a condom and hormonal contraception, respectively. Only 3.1% of the women used other types of birth control methods, such as spermicides, diaphragm, cervical cap or sterilization, intrauterine device (IUD), and natural (abstinence, fertility awareness method (FAM), and withdrawal).

### Diagnosis of vaginal infections in the study population

In the present study, participants were diagnosed with healthy or normal microbiota (66.7%), intermediate microbiota (10.4%), and vaginal infections (22.9%). However, from the women with vaginal infections, most women had aerobic vaginitis (11.8%), followed by women with bacterial vaginosis (5.6%) and candidiasis (1.7%). Only 3.9% of the women showed coinfections (see Table 1).

**Figure 1:**
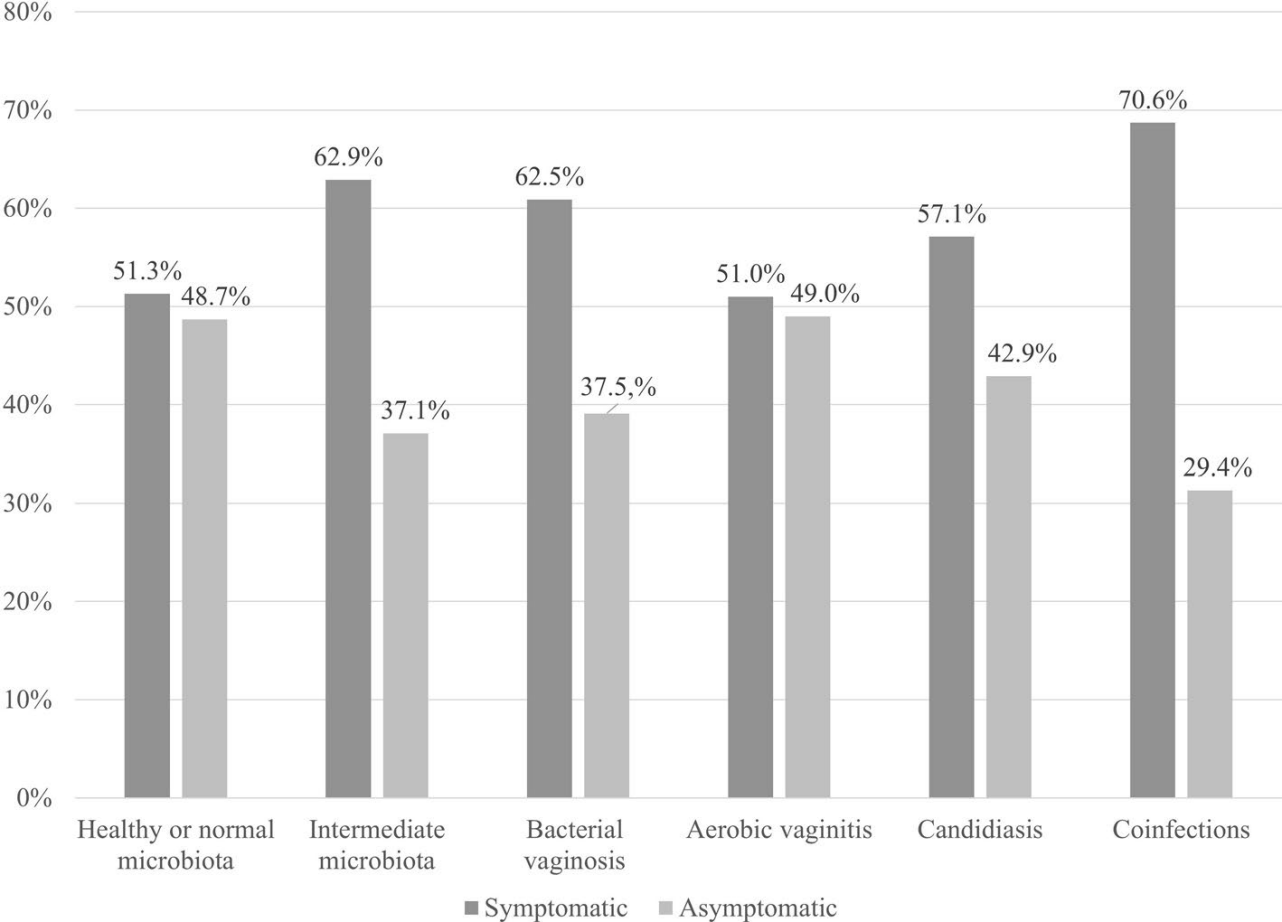
Symptomatic and asymptomatic women in this study with healthy or normal microbiota, intermediate microbiota, candidiasis, bacterial vaginosis, aerobic vaginitis, and coinfections. Legend: The Chi-square test was used to evaluate associations between symptomatic and asymptomatic women in each type of vaginal microbiota in this study. A value of *P* < 0.05 and 95% confidence intervals were considered significant for the test. All types of microbiota showed *P* ˃ 0.05 values between symptomatic and asymptomatic women; no statistically significant differences with Chi-square tests were found between symptomatic and asymptomatic women of any vaginal microbiota.

From the 95 vaginal infections, 16 volunteers showed coinfections, which four women were asymptomatic, and one woman had simultaneously three vaginal infections. The remaining 79 women were diagnosed with only one type of vaginal infection, where 41 study participants (51.9%) reported symptoms. No statistically significant difference was found between asymptomatic and symptomatic women among different types of vaginal microbiota (see Fig. 1).

Univariable logistic regression analyses were performed in all women of the present study between sociodemographic or behavioral variables and women with each type of vaginal microbiota (see Supplementary Table 3). The main statistically significant values obtained in univariable logistic regression analyses were summarized in Table 2. In our study set, unprofessional women showed a reverse odd of healthy microbiota (*P* = 0.020, OR = 0.38, 95% CI = 0.17–0.86) but women having a sexual partner (*P* = 0.020, OR = 1.64, 95% CI = 1.08–2.47) and using hormonal contraception (*P* = 0.040, OR = 2.03, 95% CI = 1.03–3.99) showed an increased odds of having healthy microbiota. However, when we applied Benjamini–Hochberg (BH) adjustment to detect false discovery rate (FDR) in multiple comparisons, only women having sexual partner remained with a statistically significant association with healthy microbiota. In opposition, women having sexual partner (*P* = 0.015, OR = 0.45, 95% CI = 0.24–0.86) and using contraceptives (*P* = 0.003, OR = 0.38, 95% CI = 0.20–0.72) demonstrated a reverse odd of having intermediate microbiota. In fact, women using condom (*P* = 0.008, OR = 0.31, 95% CI = 0.13–0.74) and hormonal contraception (*P* = 0.021, OR = 0.18, 95% CI = 0.04–0.77) showed a greater reverse odds of having intermediate microbiota, as shown in Table 2. All these correlations maintained their statistically significant association with intermediate microbiota after BH adjustment.

**Table 2:** Univariable logistic regression analyses of the main statistical values between sociodemographic or behavioral variables among women and each type of vaginal microbiota evaluated in this study.

Type of vaginal microbiota	Sociodemographic or behavioral variables	*P*-value	OR	95% CI	Adjusted *P*-value
Healthy microbiota	**Occupation**				
	Unprofessional	0.020*	0.38	0.17–0.86	0.060
	**Sexual partner**				
	Having	0.020*	1.64	1.08–2.47	0.020*
	**Birth control methods**				
	Hormonal contraception	0.040*	2.03	1.03–3.99	0.160
Intermediate microbiota	**Sexual partner**				
	Having	0.015*	0.45	0.24–0.86	0.015*
	**Contraceptive use**				
	Yes	0.003**	0.38	0.20–0.72	0.003**
	**Birth control methods**				
	Condom	0.008**	0.31	0.13–0.74	0.032*
	Hormonal contraception	0.021*	0.18	0.04–0.77	0.042*
Aerobic vaginitis	**Age**				
	Over 50	0.026*	4.46	1.20–16.66	0.104
Coinfections	**Occupation**				
	Unprofessional	0.026*	4.88	1.21–19.63	0.078
	**Civil status**				
	Free union	0.000***	16.65	3.63–76.28	0.000***

Univariable logistic regression analyses were conducted to examine how different subcategories (independent variables) were associated with the presence of each type of vaginal microbiota (dependent variables) in each category. The following subcategories of each category were used as reference for statistical analysis: Under 20 in Age; University in Education Level; Student in Occupation; Single in Civil Status; Not Having in Sexual Partner; No in Contraceptive Use; None or Don’t answer in Birth Control Methods. Odds ratios (OR) and 95% confidence intervals (CI) obtained as measurements of relative risks and the assessments of independent risk factors for vaginal infection establishment. A value of *P* < 0.05 and 95% confidence intervals were considered significant for the test: *P ≤ 0.05; **P ≤ 0.01; ***P ≤ 0.001. *N/d* non-determined. All initial values of *P* < 0.05 obtained by univariable logistic regression analyses were then evaluated through Benjamini–Hochberg (BH) adjustment to detect false discovery rate (FDR) for conducting multiple comparisons. These *P*-values evaluated by BH adjustment were illustrated in the table as adjusted *P*-values.

Only 14 of 23 women with BV (62.5%) showed symptomatic infection (see Fig. 1) demonstrating physical symptoms, such as irritation, homogeneous, and gray discharge thin with a fishy odor. Most BV women were between 21 and 30 years old (60.9%). The highest percentages of BV were also identified in student women (82.6%) and single (87.0%). However, no statistically significant differences were found among BV women in sociodemographic or behavioral variables (see Table 2). Among AV infection, 25 of 49 women (51.0%) reported symptoms in our study survey, complaining about inflammation and a yellow discharge with foul or rotten odor. Likewise, most AV women were between 21 and 30 years old (53.1%), student (67.3%) and single (83.7%). We initially found statistically significant differences in women over 50 years old (*P* = 0.026, OR = 4.46, 95% CI = 1.20–16.66), but this correlation lost statistical significance after BH adjustment. Next, VC was diagnosed in seven women where only four women (57.1%) had physical symptoms, such as pruritus and thick discharge with a color of between white to yellow. All VC cases were detected in women under 41 years old, where 5 of the 7 cases (71.4%) were between 21 and 30 years old, students and that did not have a sexual partner (see Table 1). We found no statistically significant differences between sociodemographic or behavioral variables (see Table 2). Finally, 16 cases of coinfections were diagnosed among women, only 5 women showed asymptomatic infections (29.4%; see Fig. 1). Univariable logistic regression analysis initially showed an increased odds of coinfections among unprofessional women (*P* = 0.026, OR = 4.88, 95% CI = 1.21–19.63) and women in free union (*P* = 0.000, OR = 16.65, 95% CI = 3.63–76.28). However, only women in free union (defined as couples living together for at least 3 years without being married) maintained their correlation with coinfections after BH adjustment.

When analyzing sociodemographic factors or behavioral variables simultaneously associated with all vaginal infections, we found that only unprofessional women (*P* = 0.048, OR = 2.33, 95% CI = 1.01–5.37) shared an increased odds of having any vaginal infection but lost its statistical significance after BH adjustment (see Table 3).

**Table 3:** Sociodemographic factors or behavioral variables associated with the presence of vaginal infection.

	*P*-value	OR	95% CI	Adjusted *P*-value
**Age**				
21–30	0.355	0.77	0.44–1.35	0.549
31–40	0.919	0.96	0.41–2.26	0.919
41–50	0.412	0.57	0.15–2.16	0.549
Over 50	0.181	2.39	0.67–8.59	0.549
**Education level**				
≤ Basic	0.628	0.58	0.06–5.27	0.628
Secondary	0.544	0.84	0.47–1.48	0.628
**Occupation**				
Housewife	0.639	1.49	0.28–7.85	0.639
Unprofessional	0.048*	2.33	1.01–5.37	0.144
Professional	0.556	1.20	0.66–2.18	0.639
**Civil status**				
Free union	0.143	2.72	0.71–10.37	0.215
Married	0.348	0.70	0.33–1.49	0.348
Divorced	0.143	2.72	0.71–10.37	0.215
**Sexual partner**				
Having	0.412	0.82	0.52–1.31	0.412
**Contraceptive use**				
Yes	0.603	1.13	0.71–1.82	0.603
**Birth control methods**				
Condom	0.162	1.47	0.86–2.52	0.648
Hormonal contraception	0.752	0.89	0.43–1.85	0.901
Combined	0.470	0.74	0.33–1.67	0.901
Others	0.901	1.09	0.28–4.17	0.901

Univariable logistic regression analyses were conducted to examine how different subcategories (independent variables) were associated with the presence of vaginal infection (dependent variable). The following subcategories of each category were used as reference for statistical analysis: Under 20 in Age; University in Education Level; Student in Occupation; Single in Civil Status; Not Having in Sexual Partner; No in Contraceptive Use; None or Don’t answer in Birth Control Methods. Odds ratios (OR) and 95% confidence intervals (CI) obtained as measurements of relative risks and the assessments of independent risk factors for vaginal infection establishment. A value of *P* < 0.05 and 95% confidence intervals were considered significant for the test: *P ≤ 0.05. All initial values of *P* < 0.05 obtained by univariable logistic regression analyses were then evaluated through Benjamini–Hochberg (BH) adjustment to detect false discovery rate (FDR) for conducting multiple comparisons. These *P*-values evaluated by BH adjustment were illustrated in the table as adjusted *P*-values.

### Vaginal colonization of pathogenic and opportunistic species

The vaginal colonization of each type of microbiota by pathogenic and opportunistic species was then evaluated through PCR identification of the main key species from each type of vaginal infection, specifically: *A. vaginae*, *Gardnerella* species and *M. mulieris* for BV; *E. coli* and *E. faecalis* for AV; and *C. albicans* for VC. As shown in Table 4, the most prevalent key species in vaginal colonization of the study set were *Gardnerella* species (41.8%) and *A. vaginae* (41.3%). The microbial colonization was then followed by *E. coli* (12.3%), *M. mulieris* (3.1%), *E. faecalis* (1.7%), and *C. albicans* (1.7%).

**Table 4:** Molecular detection of the main opportunistic pathogenic species among women in this study.

	Healthy microbiota *N* (%)	Intermediate microbiota *N* (%)	Bacterial vaginosis *N* (%)	Aerobic vaginitis *N* (%)	Candidiasis *N* (%)	Coinfections *N* (%)	Total *N* (%)	*P* (X^2^)
Total incidence	276 (66.7)	43 (10.4)	23 (5.6)	49 (11.8)	7 (1.7)	16 (3.9)	414 (100.0)	
***Atopobium vaginae***								
Absence	179 (64.9)	21 (48.8)	9 (39.1)	29 (59.2)	1 (14.3)	4 (25.0)	243 (58.7)	0.001 (22.9)
Presence	97 (35.1)	22 (51.2)	14 (60.9)	20 (40.8)	6 (85.7)	12 (75.0)	171 (41.3)	
Adjusted *P*-value	0.000***	0.198	0.075	0.941	0.032*	0.015*		
***Mobiluncus mulieris***								
Absence	275 (99.6)	42 (97.7)	19 (82.6)	45 (91.8)	7 (100.0)	13 (81.3)	401 (96.9)	0.001 (39.6)
Presence	1 (0.4)	1 (2.3)	4 (17.4)	4 (8.2)	0 (0.0)	3 (18.8)	13 (3.1)	
Adjusted *P*-value	0.000***	0.746	0.000***	0.048*	0.746	0.000***		
***Gardnerella spp.***								
Absence	181 (65.6)	20 (46.5)	5 (21.7)	28 (57.1)	2 (28.6)	5 (31.3)	241 (58.2)	0.001 (28.5)
Presence	95 (34.4)	23 (53.5)	18 (78.3)	21 (42.9)	5 (71.4)	11 (68.8)	173 (41.8)	
Adjusted *P*-value	0.000***	0.131	0.000***	0.872	0.131	0.052		
***Escherichia coli***								
Absence	250 (90.6)	36 (83.7)	20 (87.0)	37 (75.5)	5 (71.4)	15 (93.8)	363 (87.7)	0.038 (11.8)
Presence	26 (9.4)	7 (16.3)	3 (13.0)	12 (24.5)	2 (28.6)	1 (6.3)	51 (12.3)	
Adjusted *P*-value	0.033*	0.541	0.913	0.033*	0.374	0.541		
***Enterococcus faecalis***								
Absence	273 (98.9)	41 (95.3)	22 (95.7)	48 (98.0)	7 (100.0)	16 (100.0)	407 (98.3)	0.510 (4.3)
Presence	3 (1.1)	2 (4.7)	1 (4.3)	1 (2.0)	0 (0.0)	0 (0.0)	7 (1.7)	
Adjusted *P*-value	0.534	0.534	0.618	0.840	0.840	0.840		
***Candida albicans***								
Absence	275 (99.6)	41 (95.3)	22 (95.7)	49 (100.0)	5 (71.4)	15 (93.8)	407 (98.3)	0.001 (39.5)
Presence	1 (0.4)	2 (4.7)	1 (4.3)	0 (0.0)	2 (28.6)	1 (6.3)	7 (1.7)	
Adjusted *P*-value	0.009**	0.224	0.328	0.328	0.000***	0.224		

*N* number of women who responded in the survey within each category; *%* assigned percentage for each classification within each category. The Chi-square test (*P* (X^2^) was used to evaluate statistical differences of the presence of each opportunistic pathogen against its absence in each type of vaginal microbiota. A value of *P* < 0.05 and 95% confidence intervals were considered significant for the test: *P ≤ 0.05; **P ≤ 0.01.; ***P ≤ 0.001. All initial values of *P* < 0.05 obtained by Chi-square analyses were then evaluated through Benjamini–Hochberg (BH) adjustment to detect false discovery rate (FDR) for conducting multiple comparisons. These *P*-values evaluated by BH adjustment were illustrated in the table as adjusted *P*-values.

The distribution of each microbial species was statistically significant different among vaginal microbiota diagnoses by Chi-square test analysis, excepting for *E. faecalis*. All statistically significant *P* values were then adjusted using BH adjustment. We obtained several statistically significant values from this initial evaluation (see Table 4). The presence of *A. vaginae* showed to be statistically different in women with healthy microbiota, VC women and women with coinfections. *A. vaginae* absence was associated with healthy microbiota and its presence with VC and coinfections. *M. mulieris* presence was statistically correlated in all vaginal dysbiosis, except for intermediate microbiota and candidiasis. While its absence was associated among women with healthy microbiota. The presence of *Gardnerella* species was statistically associated among BV women and its absence correlated among women with healthy microbiota. In addition, we found that *E. coli* and *C. albicans* were significantly absent among women with healthy microbiota. But the presence of *E. coli* was also correlated in AV women while the presence of *C. albicans* was related among VC women. However, further statistical analyses were realized to fully understand these statistically significant differences in vaginal colonization among women. Therefore, multivariable logistic regression analyses were conducted to examine how different key pathogens were simultaneously associated with each type of vaginal microbiota (see Table 5). In addition, univariable logistic regression analyses were conducted to examine how different key pathogens were commonly associated with the presence of vaginal infection (see Table 6).

**Table 5:** Multivariable logistic regression analyses of all opportunistic pathogens evaluated in this study on each type of vaginal microbiota.

Type of vaginal microbiota	Opportunistic pathogen	*P*-value	OR	95% CI	Adjusted *P*-value
Healthy microbiota	*Atopobium vaginae*	0.107	0.69	0.43–1.09)	0.161
	*Mobiluncus mulieris*	0.008**	0.06	0.01–0.49	0.048*
	*Gardnerella* spp.	0.023*	0.59	0.37–0.93	0.069
	*Escherichia coli*	0.185	0.64	0.34–1.23	0.222
	*Enterococcus faecalis*	0.698	0.73	0.14–3.68	0.698
	*Candida albicans*	0.063	0.13	0.02–1.12	0.126
Intermediate microbiota	*Atopobium vaginae*	0.484	1.28	0.64–2.55	0.654
	*Mobiluncus mulieris*	0.545	0.52	0.06–4.30	0.654
	*Gardnerella* spp.	0.245	1.50	0.76–2.99	0.654
	*Escherichia coli*	0.720	1.18	0.48–2.94	0.720
	*Enterococcus faecalis*	0.362	2.28	0.39–13.37	0.654
	*Candida albicans*	0.374	2.20	0.39–12.58	0.654
Bacterial vaginosis	*Atopobium vaginae*	0.657	1.24	0.48–3.25	0.709
	*Mobiluncus mulieris*	0.020*	4.98	1.28–19.39	0.060
	*Gardnerella* spp.	0.010**	4.16	1.40–12.35	0.060
	*Escherichia coli*	0.490	0.63	0.17–2.37	0.709
	*Enterococcus faecalis*	0.481	2.31	0.22–23.85	0.709
	*Candida albicans*	0.709	1.54	0.16–15.03	0.709
Aerobic vaginitis	*Atopobium vaginae*	0.536	0.81	0.41–1.59	0.999
	*Mobiluncus mulieris*	0.080	3.29	0.87–12.50	0.240
	*Gardnerella* spp.	0.800	0.92	0.47–1.80	0.999
	*Escherichia coli*	0.009**	2.81	1.29–6.12	0.054
	*Enterococcus faecalis*	0.990	0.99	0.10–9.59	0.999
	*Candida albicans*	0.999	0.00	0.00–N/d	0.999
Candidiasis	*Atopobium vaginae*	0.095	6.62	0.72–60.79	0.285
	*Mobiluncus mulieris*	0.999	0.00	0.00–Nd	0.999
	*Gardnerella* spp.	0.703	1.44	0.22–9.19	0.999
	*Escherichia coli*	0.405	2.14	0.36–12.89	0.810
	*Enterococcus faecalis*	0.999	0.00	0.00–N/d	0.999
	*Candida albicans*	0.007**	17.94	2.22–145.11	0.042*
Coinfections	*Atopobium vaginae*	0.056	3.34	0.97–11.54	0.168
	*Mobiluncus mulieris*	0.033*	5.48	1.15–26.18	0.168
	*Gardnerella* spp.	0.359	1.76	0.52–5.94	0.431
	*Escherichia coli*	0.229	0.27	0.03–2.26	0.431
	*Enterococcus faecalis*	0.999	0.00	0.00–N/d	0.999
	*Candida albicans*	0.296	3.50	0.33–36.64	0.431

Legend: Multivariable logistic regression analyses were conducted to examine how different opportunistic pathogens (independent variables) were simultaneously associated with each type of vaginal microbiota diagnosis (dependent variables). Each type of vaginal infection (BV, VC, AV, and co-infection), healthy microbiota, and intermediate microbiota were considered categorical variables (dependent variables) for testing against different opportunistic pathogens (independent variables) detected in each type of vaginal microbiota. A value of *P* < 0.05 and 95% confidence intervals were considered significant for the test: *P ≤ 0.05; **P ≤ 0.01. *N/d* non-determined. All initial values of *P* < 0.05 obtained by multivariable logistic regression analyses were then evaluated through Benjamini–Hochberg (BH) adjustment to detect false discovery rate (FDR) for conducting multiple comparisons. These *P*-values evaluated by BH adjustment were illustrated in the table as adjusted *P*-values.

**Table 6:** Association between the presence of each opportunistic pathogen evaluated in this study on vaginal infection.

Opportunistic pathogen	*P*-value	OR	95% CI	Adjusted *P*-value
*Atopobium vaginae*	0.003**	2.03	1.28–3.23	0.006**
*Mobiluncus mulieris*	0.000***	20.76	4.51–95.45	0.000***
*Gardnerella* spp.	0.000***	2.34	1.47–3.73	0.000***
*Escherichia coli*	0.027*	2.03	1.08–3.79	0.041*
*Enterococcus faecalis*	0.722	1.35	0.26–7.08	0.722
*Candida albicans*	0.047*	4.63	1.02–21.06	0.056

Univariable logistic regression analyses were conducted to examine how different opportunistic pathogens (independent variables) were associated with the presence of vaginal infection (dependent variable). The absence of each pathogen was used as reference for this statistical analysis. Odds ratios (OR) and 95% confidence intervals (CI) obtained as measurements of relative risks and the assessments of independent risk factors for vaginal infection establishment. A value of *P* < 0.05 and 95% confidence intervals were considered significant for the test: *P ≤ 0.05; **P ≤ 0.01; ***P ≤ 0.001. All initial values of *P* < 0.05 obtained by univariable logistic regression analyses were then evaluated through Benjamini–Hochberg (BH) adjustment to detect false discovery rate (FDR) for conducting multiple comparisons. These *P*-values evaluated by BH adjustment were illustrated in the table as adjusted *P*-values.

### Factors associated with the presence of vaginal infection

Using multivariable logistic regression analyses, *M. mulieris* (*P* = 0.008, OR = 0.06, 95% CI = 0.01–0.49) and *Gardnerella* species (*P* = 0.023, OR = 0.59, 95% CI = 0.37–0.93) showed a reverse odds of healthy microbiota (see Table 5). However, only *M. mulieris* maintained its statistically significance after BH adjustment. The remaining key pathogens also showed reverse odds of healthy microbiota, but no statistically significant values were achieved. Also, none of the key pathogen showed statistically significant value in women with intermediate microbiota. Although *M. mulieris* (*P* = 0.020, OR = 4.98, 95% CI = 1.28–19.39) and *Gardnerella* species (*P* = 0.010, OR = 4.16, 95% CI = 1.40–12.35) initially showed an increased odds of BV in women, these statistical significances were lost after BH adjustment. *E. coli* (*P* = 0.009, OR = 2.81, 95% CI = 1.29–6.12) was the only key pathogen that was associated with an increased odds of AV in women but also lost its statistical correlation after BH adjustment. While *C. albicans* (*P* = 0.007, OR = 17.94, 95% CI = 2.22–145.11) showed an increased odds of VC in women even after BH adjustment. Finally, only *M. mulieris* (*P* = 0.033, OR = 5.48, 95% CI = 1.15–26.18) statistically demonstrated an increased odds of coinfections in women but also lost its statistical correlation after BH adjustment. It is important to mention that *Enterococcus faecalis* was the only opportunistic pathogen without a statistically significant value against any type of vaginal microbiota.

Furthermore, when analyzing the presence of opportunistic pathogens on women with any vaginal infection, all opportunistic pathogens were statistically associated in women with vaginal infection, excepting for *E. faecalis* (see Table 6). *M. mulieris* (*P* = 0.000, OR = 20.76, 95% CI = 4.51–95.45) was the most prominent analyzed key pathogen among women with a vaginal infection, followed by *C. albicans* (*P* = 0.047, OR = 4.63, 95% CI = 1.02–21.06) and *Gardnerella* species (*P* = 0.000, OR = 2.34, 95% CI = 1.47–3.73). Finally, both *A. vaginae* (*P* = 0.003, OR = 2.03, 95% CI = 1.28–3.23) and *E. coli* (*P* = 0.027, OR = 2.03, 95% CI = 1.08–3.79) showed a similar increased odds of having a women with vaginal infection. After BH adjustment, *C. albicans* was the only pathogen that did not maintain its statistical significance among women with vaginal infection.

## Discussion

To the authors’ knowledge, this is the first epidemiologic analysis done in Ecuador to evaluate the prevalence of key pathogens involved in the etiology of the different types of vaginal infections described in the literature. Similar to previous studies, healthy vaginal microbiota was identified in two-thirds of the volunteers (66.7%)^13,32,33^. In 2002, Cauci et al.^34^ conducted an epidemiological analysis of healthy microbiota, intermediate microbiota and BV infection on Italian women. These authors found 67.8% of healthy microbiota in peri and postmenopausal women with a mean age of 45.3 years old. Other countries have shown different rates of healthy vaginal microbiota, such as the USA (60.6%), Chile (58.3%), and Turkey (47.7%)^35–37^.

Only 10.4% of the participants displayed intermediate microbiota in our study set. Similar results were also obtained in other studies^36,38,39^. However, several studies have reported higher rates of intermediate microbiota in women^44–46^, showing rates between 36.3 and 69.2%. Nonetheless, in 2002, Cauci et al.^34^ reported a lower rate of intermediate microbiota (6.1%) when compared to this study. It is important to mention that an intermediate microbiota is different from a normal and healthy microbiota, being characterized by a substantial reduction of lactobacilli^40^. It is postulated to be an independent pathological condition or a temporary transition to a vaginal infection (such as BV, AV, and VC)^4^. However, it is still not classified as a full or defined type of infection. It is relevant to mention that this intermediate microbiota may also go back to a normal and healthy microbiota^41^. As reported by many authors, the composition of the vaginal microbiome can vary throughout a woman’s life in response to endogenous and exogenous factors^41–43^. So, future longitudinal studies should be conducted in order to identify sociodemographic or behavioral factors that could contribute to different outcomes from this type of microbiota.

In our study population, vaginal infection was diagnosed in 95 women (22.9%) at a similar rate to the prevalence reported in the USA (28%)^47^ but lower than the prevalence detected in Syria (51%)^48^. One of the highest prevalence of vaginal infection was reported in Bosnia (96%)^3^. Moreover, most of the participants with one well-established infection in the present study were below the age of 31 years old. Nevertheless, other studies identified a higher risk of vaginal infection in women older than 30 years old^49,50^. In this study, no particular age group was associated with a greater risk of vaginal infection.

In this study, we found that women in free union had a statistically higher odds of coinfections. Other studies reported no apparent association between marital status or long term relationship and the presence of infection^49,51^. However, in Hainan (an island province of China), a study done in 2014 by Na and colleagues reported that marriage was significantly associated with a higher risk of VC in their group set (689 cases and 652 controls)^50^. The present study did not show a clear association of sociodemographic or behavioral variables among BV and VC women. Although previous studies reported a higher prevalence of vaginal infection in women with a lower level of education^49,50^, this study did not show any statistically significant correlation between education level and women with vaginal infection after adjusting *P*-values (see Tables 2 and 3). Also, several studies reported a negative association between BV infection and the use of condoms^52–55^. In this study, women having a sexual partner and women using contraceptive (condom and hormonal contraception) showed a reverse association among women with intermediate microbiota (see Table 2). In addition, women having a sexual partner showed an increased odds of having healthy microbiota. These behavioral variables could also prevent lactobacilli reduction usually associated with intermediate microbiota and consequently avoid future vaginal infection. However, no straightforward association was observed between these behavioral variables and any type of vaginal infection in women. In agreement, other studies also revealed that the use of oral contraceptives, intrauterine device, and barrier methods were not related to the risk of vaginal infection^53,55,56^. It is important to remember that intrinsic and extrinsic factors, such as age, ethnicity, menstruation cycle, lifestyle habits, use of contraceptives and antibiotics may have an impact on the vaginal microbiota^57–61^. Associations between the microbiota plus background variables and clinical outcomes in different stages of a woman’s life are complex, and which intrinsic or external factors drive the community composition it is not fully understood^61,62^. Behavioral factors, such as sexual behavior and methods of birth control, may contribute to lactobacilli reduction and leading to an intermediate microbiota or even a vaginal infection^54,63–65^. The present study showed the results of the vaginal microbiota colonization in Ecuadorian women from Pichincha province (considered as another ethnicity factor), after controlling the confounding factors, such as sociodemographic, behavioral or environmental variables. Most of the population set was characterized as mestizo ethnicity. Further studies should evaluate a more profound impact of host socioeconomic or educational factors on vaginal microbiota, as previous epidemiological studies in other countries^61,66,67^.

The most prevalent form of vaginal infection in our study was AV (51.6%; 49 of 95 infection cases), followed by BV (24.2%; 23 of 95 infection cases) and then VC (7.4%; 7 of 95 infection cases). Although few studies analyzed the presence of different types of vaginal infection in women, Jahic and colleagues^3^ reported in 2013 a similar prevalence of AV (51%) in their population set, a lower rate of BV (15.0%) and a higher rate of VC (17.0%). Another study realized by Mulu et al.^68^ showed a more similar candidiasis rate (9.2%) as the present study.

Aerobic vaginitis was first characterized in 1999 and then in 2002 by Donders and colleagues in Belgium^42,69–71^. Little is still known about its global epidemiology and implications, when comparing BV and VC. Although our AV prevalence was similar to a study reported in Bosnia (51%)^3^, other countries showed lower AV prevalence in their studies, such as Belgium (7.9% and 10.0%)^71,72^, Brazil (4.9%)^73^, and USA (8–11.0%)^42^. This low prevalence of AV had been reported in several review studies^42,71,74^. Besides, Tansarli and colleagues^74^ reported a prevalence of 5–10.5% of symptomatic AV women, while Kaambo and colleagues^42^ showed a rate of AV between 8.0 and 11.0% in pregnant women and also 5–24.0% of AV in women with symptomatic infection. In our population set, 49% of AV infection was diagnosed in asymptomatic women showing a smaller prevalence when compared to another study realized by Gondo et al.^45^ in Brazil, where 57.1% of AV was detected in asymptomatic women.

BV prevalence was 24.2% in the present study, and similar to other studies in Ecuador^75^ (31.5%), Perú^76^ (27.0%), and the USA^77^ (29.2%). Some countries of Europe demonstrated a lower prevalence of BV in their study sets of pregnant women^10,78^, such as France (7.1%) and Portugal (3.88%). While other studies described a higher BV prevalence of 48.6% in Ethiopia^79^ and 44.8% in India^80^. Most epidemiological studies have demonstrated a variety of BV prevalence accordingly to their geographical locations^49^. This variety of BV prevalence has also been reported in several review studies^18,54,81^, where it had been normally reported a BV prevalence between 6.1% and 51.6%. Finally, in the present study, 62.5% of women with BV were classified as symptomatic infection, showing a similar prevalence as previously reported by Gondo and colleagues^45^ in Brazil (66%). However, in the USA, Koumans and colleagues^77^ stated only 15.7% of women symptomatic with BV.

VC was identified only in 7.4% of women with vaginal infection in the present study, showing a similar prevalence to a study realized in Ethiopia (8.3%)^68^. However, several countries reported a higher rate of candidiasis, more precisely, Brazil (52.4%), Italy (43.5%), India (35.0%), Nigeria (36.0%), Chile (43.9%) and USA (20–30.0%)^35,82–87^. In our group set, 57.1% of VC were diagnosed in symptomatic women, demonstrating a higher prevalence when compared to Mulu et al.^68^ (6.8%) and lower prevalence when compared to Gondo et al.^45^ (92.0%). Most studies reported the presence or absence of infection, as well as their pathogen colonization; however, little is known about the epidemiological prevalence of symptomatic and asymptomatic women until now.

Concerning coinfections, we detected 16 cases from a total of 95 vaginal infections, where 70.6% of women had symptomatic infection. Despite this high percentage of symptomatic coinfections, another study revealed a higher prevalence of symptomatic infection in the presence of coinfections^45^, more precisely 85.7%. Also, Rivers and colleagues^88^ showed a high prevalence of symptoms (80% of abnormal vaginal discharge) in women with a coinfection for BV and candidiasis. This work supported the findings of previous studies by reporting a more significant number of symptomatic women with multiple vaginal infections. However, further studies are necessary to analyze asymptomatic women in each type of vaginal infection. Finally, it is important to mention that the only coinfection simultaneously diagnosed with BV, AV, and candidiasis was reported in a woman with several sexual partners. Although it was not possible to establish any statistical significance, this coinfection seems to indicate that several sexual partners could be a risk factor, as already reported in several previous studies^54,89^.

Further analysis was done in this study to identify the main key species commonly associated with each vaginal infection. This analysis was then compared with previous studies of other countries, as shown in Table 7. Although AV was the main diagnosed infection in our study, only 24.5 and 2.0% of these infections were colonized by *E. coli* and *E. faecalis*, respectively, demonstrating an AV dysbiosis induced by other pathogenic and/or opportunistic species in these Ecuadorian women. These results differ from previous reports that showed a higher prevalence of *E. coli* and *E. faecalis*^90,91^. Studies from Bosnia and Italy showed a prevalence of *E. coli* between 55.0 and 86.7% and *E. faecalis* between 40.0 and 52.0%. Moreover, Von Gruenigen and colleagues^92^ identified rates of 28.0 and 44.0% of *E. coli* and *E. faecalis*, respectively, in their small population set in the USA. In Japan, Puapermpoonsiri and colleagues^33^ reported a prevalence of 38.0% of *E. faecalis* in their study set. However, other studies done in developing countries, such as Nigeria, Mexico, and Iraq, detected a similar or less prevalence of *E. coli* in AV women^93–95^, more precisely, 16.2, 13.5 and 6.0%, respectively. In Greece, Iavazzo and colleagues^96^ reported a lower prevalence of *E. coli* and *E. faecalis* in a large population set (1.632 women), more precisely, 4.0 and 0.3%, respectively. It is important to mention that several studies have described other AV-associated aerobes than *E. coli* and *E. faecalis*^74,93,96^, such as *Streptococcus* and *Staphylococcus* species. This data could explain the low values of *E. coli* and *E. faecalis* prevalence found in our study. More research is needed to ascertain other species related to vaginal infections^93–95^.

**Table 7:** Summary of vaginal infection studies in women (including this study).

	Population description	Study group (n)	Country	Methodology	Microorganism species detected (%)						References
					*A. vaginae*	*Gardnerella* spp.	*M. mulieris*	*E. coli*	*E. faecalis*	*C. albicans*	
**Bacterial vaginosis**											
1	Women in reproductive age (age range 18–56)	414	Ecuador	Microscopic examination, Nugent criteria, PCR	60.9	78.3	17.4	13.0	4.3	4.3	This study
2	Pregnant teenage (age range 10–19)	95	Ecuador	PCR	100.0	93.7	35.7	Na	Na	Na	^20^
3	Women (age 15–54)	223	Brazil	Multiplex PCR	9.3	45.7	3.7	Na	Na	Na	^97^
4	Premenopausal women (age 18–48)	196	USA	Microscopic examination and PCR	Na	53.0	Na	Na	Na	Na	^127^
5	Women (age range 14–37)	50	USA	Clinical examination and PCR	54.0	Na	Na	Na	Na	Na	^98^
6	Pregnant women (age 19–41)	206	Portugal	PCR	Na	67.4	Na	Na	Na	Na	^10^
7	Women (age 22–53)	116	Lithuania	Clinical and microscopic examination, PCR	89.7	100.0	Na	Na	Na	Na	^99^
8	Women (age 16–45)	538	Bulgaria	Multiplex PCR	68.1	98.4	17.0	Na	Na	Na	^100^
9	Postmenopausal women (mean 55.6 ± 2.6 years)	52	China	16S rRNA PCR	65.5	82.8	Na	Na	Na	Na	^102^
10	Premenopausal women (age 18–48)	196	China	Microscopic examination and PCR-DGGE	17.1	63.2	Na	Na	Na	Na	^101^
**Aerobic vaginitis**											
1	Women in reproductive age (age range 18–56)	414	Ecuador	Microscopic examination, Nugent criteria, PCR	40.8	42.9	8.2	24.5	2.0	0.0	This study
11	Women with gynecologic cancer (age Na)	26	USA	Microscopic examination and culture	Na	Na	Na	28.0	44.0	Na	^92^
12	Pregnant women (age 15–40)	326	Japan	Microscopic examination and culture	Na	100.0	13.0	Na	38.0	25.0	^33^
13	Women (age 18–45)	100	Bosnia	Clinical examination and culture	Na	Na	Na	55.0	52.0	17.0	^3^
14	Women with a diagnosis of AV (mean age 33.5 ± 8.68 years)	81	Italy	Clinical examination and culture	Na	Na	Na	86.7	40.0	Na	^91^
15	Cervical discharge specimens (age Na)	6811	México	Microscopic examination and culture	Na	Na	Na	13.46	Na	Na	^94^
16	Symptomatic women (age range 18–57)	1632	Greece	Microscopic examination, culture and API 20 methods	Na	40.4	Na	4.0	0.3	42.5	^96^
17	Women (age range 15–50)	250	Nigeria	Microscopic examination and culture	Na	Na	Na	6.0	Na	Na	^93^
18	Non-pregnant women (age Na)	80	Iraq	Microscopic examination an biochemical test	Na	Na	Na	16.2	Na	Na	^95^
**Candidiasis**											
1	Women in reproductive age (age range 18–56)	414	Ecuador	Microscopic examination, Nugent criteria, PCR	85.7	71.4	0.0	28.6	0.0	28.6	This study
19	Adolescents (age 13–17)	213	Ecuador	Microscopic examination	Na	Na	Na	Na	Na	23.7	^75^
20	Adolescents (age 10–19)	100	Brazil	Microscopic examination and culture	Na	Na	Na	Na	Na	22.0	^103^
21	Women with candidiasis (age 14–51)	150	Colombia	Microscopic examination and culture	Na	Na	Na	Na	Na	80.0	^104^
22	Women with candidiasis (age range 15–94)	951	Italy	Culture	Na	Na	Na	Na	Na	77.1	^85^
23	Women with diagnosis of candidiasis vulvovaginal (age Na)	77	Belgium	PCR	Na	Na	Na	Na	Na	78.6	^105^
24	Pregnant women (age 18–30)	1163	Malaysia	Microscopic examination and culture	Na	Na	Na	Na	Na	17.2	^106^
25	Women (age 21–29)	100	Nigeria	Culture	Na	Na	Na	Na	Na	36.0	^86^
26	University students (age range 18–41)	50	Ghana	Culture	28.0	Na	Na	Na	Na	22.0	^107^

*Na* not analyzed.

*Gardnerella* spp. was the most frequent pathogenic species in BV infection (78.3%), followed by *A. vaginae* (60.9%) and finally by *M. mulieris* (17.4%). *M. mulieris* and *Gardnerella* species significantly increased the odds for BV infection. However, no statistically significant association was found between *A. vaginae* and BV, and after BH adjustment neither *Gardnerella* spp. nor *M. mulieris*. These results were below the prevalence of *G. vaginalis*, *A. vaginae* and *M. mulieris* identified in our previous study done in pregnant teenagers^20^. One plausible explanation could be due to the higher number of volunteers and adult women (age range 18–56 years old) in the present study. Nevertheless, when compared to other Latin America countries (such as Brazil), the prevalence of the three BV-associated anaerobes maintained the same vaginal colonization dominance but with higher percentages of detection^97^, more precisely: *Gardnerella* species (78.3% *versus* 45.7%); *A. vaginae* (60.9% *versus* 9.3%); and *M. mulieris* (17.4% *versus* 3.7%). In the USA, Schwebke and colleagues^98^ detected *A. vaginae* in similar prevalence colonization (54.0%) when compared to our study (55.4%). Likewise, several studies conducted in Europe (such as Lithuania^99^ and Bulgaria^100^) reported a higher prevalence of the same BV-associated anaerobes, but maintaining the same hierarchy order (see Table 7). Finally, in China, two studies demonstrated a similar prevalence of *Gardnerella* species and *A. vaginae* in their group sets^101,102^, more precisely, 63.2–82.8% and 17.1–65.5%, respectively. These prevalence values and hierarchy order of anaerobes in BV could appoint to a co-dependence of *A. vaginae* and *M. mulieris* in the vaginal colonization after an initial growth or biofilm establishment by *Gardnerella* species, as postulated by several authors.

VC was the least vaginal infection diagnosed in this study, and only 28.6% of these cases had *C. albicans* as part of the vaginal microbiota dysbiosis. Our results differ from some studies worldwide (Colombia, Italy and Belgium) but in agreement with most studies, as shown in Table 7. In 2010, Vaca and colleagues^75^ reported a prevalence of 23.7% of *C. albicans* in their study set of adolescents between 13 and 17 years old in Ecuador. In other Latin-American countries, such as Brazil and Colombia, *C. albicans* prevalence in VC also fluctuated between 22.0 and 80.0%^103,104^, respectively. In Europe countries (Italy and Belgium), studies reported a more constant and prevalent existence of *C. albicans* in VC^85,105^, more precisely, around 77.1 and 78.6%. In opposite, Masri and colleagues^106^ reported a prevalence of 17.2% of *C. albicans* in pregnant women from Malaysia. Olowe and colleagues^86^ showed a prevalence of 36% of *C. albicans* (36%) in pregnant women while Aubyn and Tagoe^107^ reported only 22.0% of *C. albicans* in Ghana. These findings suggest the possibility of other *Candida* species being responsible for VC, as proposed by several previous studies^35,83,85,105^, such as *C. parapsilosis*, *C. tropicalis*, *C. krusei* and *C. glabrata*^108–110^.

In summary, previous studies support the results obtained in our study among Ecuadorian women. The present study identified AV as the leading cause of vaginal infections in our population set. The major findings were the associations obtained between several key pathogens and the different types of vaginal microbiota through univariable and multivariable logistic regression analyses. Univariable logistic regression analysis showed a positive correlation between the presence of vaginal infection and all key pathogens, except for *E. faecalis* and *C. albicans*. Furthermore, multivariable logistic regression analysis showed the possibility to use certain key pathogens as microbial predictors for different types of vaginal infections. More exactly, *Gardnerella* species and *M. mulieris* were negative correlated with healthy microbiota but positively associated with BV women. Only *M. mulieris* was correlated with coinfections. Only *E. coli* was statistically associated with AV women, but it was identified in a low percentage of AV women, indicating a plausible association of AV with other species, such as *Staphylococcus* or *Streptococcus* species. Also, *C. albicans* was correlated with VC women but it was only detected in 28.6% of the cases, suggesting the eventual involvement of other *Candida* species in the establishment of this infection. It is important to note that several significant *P*-values were reduced in data analysis after Benjamini–Hochberg (BH) adjustment, which we used to detect false discovery rates in multiple comparisons. However, even with BH adjustment, both logistic regression analyses appointed *M. mulieris* as the strongest microbial key pathogen among women with vaginal infection. These results suggest the possibility to use *M. mulieris* as a potential key predictor for vaginal infections. Several studies have already postulated the utility of the microbial composition and the presence of certain microorganisms as key predictors of increased risk to develop vaginal infections^111–113^. Further studies should be conducted to evaluate the longitudinal association of vaginal microbiota and quantification of certain key pathogens. To the authors' knowledge, this is the first study on Ecuadorian women to simultaneously assess the prevalence of several types of vaginal infection and opportunistic key pathogens.

However, there are some major limitations of the present study: (1) it is a cross-sectional study and therefore unable to establish temporal relationship between vaginal infections and sociodemographic or behavioral variables, and vaginal pathogens, (2) the study did not evaluate the prevalence of lactobacilli in vaginal microbiota, and (3) this study only evaluated the prevalence of a single *Candida* species. It is also important to mention that classical and molecular methods were applied with only one vaginal swab of each volunteer and no commercial kit was used for DNA extraction. Therefore, the results of the present study could lead to an underestimation of the prevalence of opportunistic pathogens or even infections in vaginal samples. Another drawback was the lack of quantitative data, which may allow us to assess the status of colonization of the distinct microbial taxa. Also, DNA sequencing of the samples could allow us to identify the species present in vaginal microbiota with better reliability and possibly analyze the clades to which each of the species belong. Further studies should be conducted in Ecuador to confirm the prevalence of several types of vaginal infections among women.

## Methods

### Study area, design, and subject selection

This study was conducted in the Microbiology Institute at the Universidad San Francisco de Quito (USFQ) from June 2016 to November 2017 according to the inclusion and exclusion criteria. Inclusion criteria were defined as following: (1) being 18 years old or older; (2) being born and raised in Ecuador; (3) menstruation ending since at least 2 days or antimicrobial treatment in the vagina within 3 months, and no sexual intercourse within 2 days before sample collection. Exclusion criteria were defined as following: (1) women who were under legal age; (2) women who were in the period of pregnancy, menstruation, or lactation; (3) women with any evidence of macroscopic cervical bleeding or known disease (e.g. immune disease, diabetes or other type of disease, and HIV or other severe infection). A total of 414 Ecuadorian women of Hispanic ethnicity and mostly in reproductive age (18 and 56 years old) volunteered to be part of the study set. The enrolled women received a kit containing an informed consent form approved by the Bioethics Committee of the USFQ and the Ministry of Health of Ecuador (Contrato Marco de Acceso a los Recursos Genéticos No. MAE-DNB-CM-2016-0046); a standardized medical survey, which included demographic, sexual and health behavior-related questions, as well as, information about clinical history (previous history of vaginal infections and their antimicrobial treatments) and possible symptoms (such as, change in color, odor or amount of vaginal discharge, vaginal itching or irritation, burning sensation or pain during intercourse and urination and even light vaginal bleeding or spotting); and a vaginal transport swab system (Stuart's transport media swabs; Copan Diagnostics Inc.), which the volunteers used to provide a vulvovaginal swab sample. The study was supervised by a physician, a psychologist, and a full-time researcher from the USFQ. All methods were performed in accordance with the relevant guidelines and regulations of the Microbiology Institute at the Universidad San Francisco de Quito (USFQ).

### Ethics statement

The study was approved by the Ethics Committee of the USFQ (Protocol code: 2016-023IN by MSP-VGVS-2016-0244-O review board) in Quito. The exclusion criteria included the absence of a legible and full disclosure survey or informed consent, a previous antimicrobial treatment in the last three months, an inadequate result in DNA extraction and/or in microscopic examination.

### Samples collection

The participants were informed by the associated physician how to collect their vulvovaginal swab sample. This sterile swab was brushed against the lateral vaginal walls to collect the fluid sample and was immediately placed in the transport media, stored at 4 °C and processed within the first 12 h. The analysis was carried out in the bacteriology laboratory of the Microbiology Institute at Universidad San Francisco de Quito (MI-USFQ), as previously realized in a study by Pacha-Herrera and colleagues^114^. The swab was used to prepare a vaginal smear for the microscopic examination of the vaginal microbiota, according to the Nugent and colleagues^26^. Briefly, each vaginal smear was obtained by rolling the swab onto a glass slide, then heat-fixed and Gram-stained by using safranin as the counterstain. Following the Gram smear procedure, the swab was placed in 2 ml of phosphate buffer saline (PBS) and shaken vigorously until the solution turned cloudy through a vortex for approximately 3 min. The remaining vaginal material was collected by centrifugation at 13,000 rpm for 5 min. The obtained pellet was suspended into two aliquots of 1 ml of saline solution (0.9% NaCl) in separated microtubes. Then, one aliquot was used for microbial growth in different medium cultures and wet mount procedure for better AV and VC diagnosis (see section “Diagnosis of vaginal microbiota”), while the other aliquot was used in the DNA extraction process (next section).

One hundred microliters of suspension were plated onto Petri dishes containing nutrient agar for less fastidious microorganisms (*Escherichia coli*, *Enterococcus faecalis*, *Staphylococcus* and *Streptococcus* sp.), 5% human blood agar (HBA) and chocolate agar (heated human blood agar) for fastidious microorganisms (*Atopobium vaginae*, *Mobiluncus mulieris* and *Gardnerella vaginalis*), Sabouraud dextrose agar (SDA) for *Candida* sp. and de Man, Rogosa and Sharpe agar (MRS agar) for *Lactobacillus* sp. The plates were incubated at 37 °C for 48 h, under anaerobic conditions, and microbial colonies were analyzed and identified by gram staining, biochemistry properties (catalase, oxidase, and hemolysis), and PCR (data not shown).

### DNA extraction

DNA extraction was developed according to previously published protocols^10^. The swab was placed in 2 ml of phosphate buffer saline (PBS) and shaken vigorously until the solution turned cloudy, through a vortex, for approximately 3 min. The vaginal cells were collected by centrifugation at 13,000 rpm for 5 min. The obtained pellet was suspended in 1 ml of saline solution (0.9% NaCl). The aliquot of 1 ml of saline solution (0.9% NaCl) was incubated at 100 °C in a water bath for 15 min. After that, all samples were immediately frozen at − 20 °C for 15 min. The samples were then centrifuged at 13,000 rpm for 15 min, and the supernatant was then divided into two tubes with 500 μl volumes, one stored at − 20 °C and the other at − 80 °C. Once the extraction procedure was completed, DNA quantification was performed with a Nanovue spectrophotometer (GE Healthcare Life Science). Concentrations of DNA in ng/μl were measured, as well as the phenolic contaminants (260/230) and the protein contaminants (260/280). Finally, two aliquots of DNA, between 10–20 ng/µl, were stored for future Polymerase Chain Reaction (PCR) analysis.

### Polymerase chain reaction

PCR assays were performed with the 414 samples on a T100 Thermal Cycler (Bio-Rad, CA, USA) using primers for target genes previously used in other published studies^115–119^, such as *16S* rRNA (*Atopobium vaginae, Mobiluncus mulieris,* and *Gardnerella* species), *ddl* (*Enterococcus faecalis*), *adk* (*Escherichia coli*) and *KER1* (*Candida albicans*) genes (see Supplementary Table 1). The reactions for all bacteria were performed as singleplex PCR in a total volume of 20 µl containing 0.50 units of Go Flexi Taq polymerase, 1 × Green PCR Buffer with 2.5 mM MgCl_2_ (Promega, WI, USA), 0.2 mM of dNTPs (Promega, WI, USA), 0.5 µM of each primer and 4 μl of DNA template and the remaining volume with molecular grade H_2_O. For *Enterococcus* spp., reactions were performed as singleplex PCR in a total volume of 20 µl containing 0.50 units of Go Flexi Taq polymerase, 1 × Green PCR Buffer with 2.5 mM MgCl_2_ (Promega, WI, USA), 0.6 mM of dNTPs, 1.6 µM of each primer and 4 µl of DNA template and the remaining volume with molecular grade H_2_O. The respective use of negative (without DNA sample and samples with other related bacteria) and positive (collection of identified strains of each species through DNA sequencing) controls were used in each PCR assay. These positive controls were provided by the Microbiology Institute at USFQ. All samples were randomly performed in duplicate or triplicate with different negative and positive controls.

After PCR amplification, a volume of 4 µl from each PCR product was visualized in 1.5% (w/w) agarose (Promega, WI, USA) gel electrophoresis using 0.1% ethidium bromide staining.

As reported in our previous study^20^, positive samples of *A. vaginae* were sequenced to confirm their identity due to the lack of a strong specificity from its primers and *E. coli* were validated by API 20E strips (Biomerieux API). Meanwhile, *Candida albicans*, *G. vaginalis,* and *M. mulieris* primer sets showed a strong specificity, and their validation confirmed in previous studies (see Supplementary Table 1). The eventual confirmation step for *A. vaginae* used the following universal primers for 16S rRNA sequencing (27Fw-AGA GTT TGA TCM TGG CTC AG and 805Rw-GAC TAC CAG GGT ATC TAA TC; temperature of annealing: 62 °C) through a PCR assay carried out with a final volume of 50 μl (adapted from Salinas et al.^20^) and sent to Functional Biosciences, Inc (Madison, WI, USA). The 16S rDNA sequences were compared to known sequences in GenBank with the advanced gapped BLAST (basic local alignment search tool) algorithm.

### Diagnosis of vaginal microbiota

The vaginal sample evaluation was made according to the presence of symptoms, clinical findings during the medical survey, and by microbiological criteria result obtained by microscopy examination (Gram-stained and wet mount smears). Briefly, the recognition of vaginal infections was assessed using a set of previously defined variables (see Supplementary Table 2).

The vaginal smear was obtained by rolling a swab onto a glass slide, and then the smear was heat fixed, Gram-stained, and classified according to the Nugent Score^26^. Each smear was evaluated by 10–15 microscopic fields under oil immersion (1000 × magnification) and evaluated for several morphotypes. The samples were assigned a score of 0–10, in which the criteria for healthy or normal vaginal microbiota were 0–3, while intermediate microbiota were 4 to 6, and bacterial vaginosis were 7–10^26^. The Nugent score provides a protocol for measuring scores and gives a total summed score depending on the number of large gram-positive rods (*Lactobacillus* morphotypes), small gram-variable rods (*G. vaginalis* morphotypes), small gram-negative rods (*Bacteroides* spp. morphotypes), and curved gram-negative rods (*Mobiluncus* spp. morphotypes).

After an initial evaluation by Nugent criteria, all samples were then evaluated through their microscopic examination of wet mounts from the previous saline solution aliquot (see “Vaginal colonization of pathogenic and opportunistic species”). These wet mount preps were used for better detection of *Trichomonas vaginalis*, clue cells, aerobic vaginitis evaluation according to Schröder's classification but refined in 2005 by Donders et al.^71^ and also vulvovaginal candidiasis evaluation accordingly to Marot-Leblond et al.^120^ in 2009. Briefly, from each selected sample, a drop of the saline solution aliquot was placed onto a clean glass slide, cover with a coverslip, and firstly examined microscopically using high power (40 ×) objective for the presence of *Trichomonas vaginalis*, leukocytes and clue cells. Then, the same wet mount prep was evaluated through a phase-contrast microscope (400 × magnification) for AV and VC diagnosis. For AV, the microscopic examination in a total of ten microscopic fields included signs of the absence or low number of *Lactobacillus* morphotypes (average of < 5 cells per field), positive for cocci or coarse bacilli in high number (average of > 20 cells per field), presence of parabasal epithelial cells representing > 10% of the epithelial cells, and/or positive for leukocytes (aggravated AV diagnosis if they showed granular appearance). Aggravated AV diagnosis was defined as the most extreme form of aerobic vaginitis under Donders evaluation from Schröders classification^71^, where AV samples showed lactobacilli severely depressed or absent because of overgrowth of other bacteria (Cocci or chains), more than 10 leukocytes per epithelial cell present in the samples and more than 50% of the leukocytes had a toxic appearance on their granular appearance due to abundant lysozyme activity (‘toxic leukocytes’). Finally, VC was assessed accordingly to Marot-Leblond and colleagues through at least two of the three applicable criteria: (1) positive Gram-stain or wet mount smear preparation with yeast cells and/or pseudo hyphae in high number (average of 5 > yeast cells and/or pseudo hyphae per field) in more than two in a total of ten microscopic fields; (2) and positive culture in Chocolate and Blood Agar and/or Sabouraud dextrose agar (SDA); (3) eventual symptoms (thick, white vaginal discharge with no odor, vulvar and vaginal pruritus, burning, or dyspareunia) or clinical history (previous infection) obtained from the medical survey. Absence of yeast cells and/or pseudo hyphae or a low number of *Candida* spp. (less than 5 yeast cells and/or pseudo hyphae per field) result on Gram-stain and wet mount smears observation together with a negative growth culture was considered as normal *Candida* colonization rather than VC^120^.

### Statistical analysis

Univariable logistic regression analysis was conducted to examine how different subcategories (independent variables) were associated with the presence of each type of vaginal microbiota (dependent variables) in each category of sociodemographic or behavioral variables. The same univariable logistic regression analysis was used to evaluate the association between sociodemographic or behavioral variables and the prevalence of vaginal infection in women. The following subcategories of each category were used as reference for these statistical analyses: under 20 in Age; university in education level; student in occupation; single in civil status; not having in sexual partner; no in contraceptive use; none or don’t answer in birth control methods. Each type of vaginal infection (BV, VC, AV, and co-infection), healthy microbiota and intermediate microbiota were considered categorical variables for testing differences against demographic variables (age and civil status), socioeconomic variables (level of education and occupation) and personal habits (having sexual partner and method of birth control). The Chi-square test was used to evaluate associations between symptomatic and asymptomatic women in each type of vaginal infection in this study. The Chi-square test was also used to evaluate associations between the prevalence of vaginal infection with the presence of each opportunistic pathogen (*A. vaginae, M. mulieris, Gardnerella* spp., *E. coli*, *E. faecalis*, and *C. albicans*), when compared to its absence.

Furthermore, a multivariable logistic regression analysis was also performed to correlate the presence of multiple opportunistic pathogens (independent variables) with the outcome of each type of vaginal microbiota (dependent variable). Both logistic regression analyses calculated *P*-values, Odds Ratios (OR) and 95% Confidence Intervals (CI) for each outcome. *P*-values and Odds ratios) were applied for an association, as previously used in other studies^121–123^. Therefore, the *P*-value was used as a test of association, while the OR was then used as a measure of association^124^. A value of *P* < 0.05 and 95% confidence intervals were considered significant for the association test. All initial values of *P* < 0.05 obtained by univariable logistic regression, Chi-square and multivariable logistic regression analyses were then evaluated through Benjamini–Hochberg (BH) adjustment to detect false discovery rate (FDR) for conducting multiple comparisons. All statistical analyses were performed using SPSS version 25.0 (SPSS Statistics for Windows Version 25.0, Armonk, NY, IBM Corp), excepting for Benjamini–Hochberg (BH) adjustment. The BH adjustment was realized using Seed-based d Mapping software (SDM, version 6.21, https://www.sdmproject.com, formerly “Signed Differential Mapping”)^125,126^.

## Supplementary information

Supplementary Tables.
