# Supplementary material for: Vaginal microbiota evaluation and prevalence of key pathogens in ecuadorian women: an epidemiologic analysis
Source: Sci Rep. 2020 Oct 27;10:18358. doi: 10.1038/s41598-020-74655-z (PMC7591572; doi:10.1038/s41598-020-74655-z)
Supplement: Supplementary file 1 — Supplementary Tables. [file 41598_2020_74655_MOESM1_ESM.docx]

**Manuscript title: Vaginal Microbiota Evaluation and Prevalence of Key Pathogens in Ecuadorian Women: An Epidemiologic Analysis**

**Author list:** Ana María Salinas, Verónica Gabriela Osorio, David Pacha-Herrera, Juan S. Vivanco, Ana Francisca Trueba, António Machado

**Supplementary Table 1**. PCR primers and thermocycling conditions used in this study.

| **Set** | **Name** | **Sequence (5′-3′)** | **Target** | **PCR cycling conditions** | **Size of fragment** | **Target gene** | **Specificity %** | **Validation** | **Reference** |
| --- | --- | --- | --- | --- | --- | --- | --- | --- | --- |
| 1 | Atop109-Fw | GAGTAACACGTGGGCAACCT | *Atopobium vaginae* | Pre-melt phase at 94 °C for 2 min and denaturation at 94 °C for 30 s; Annealing at 62 °C for 30 s, and extension at 72 °C for 1 min (30 cycles with an additional 5 min of extension step) | 221 bp | *16S* rRNA | 16.7% | Samples sequenced to confirm identity | ^115^ |
|  | Atop109-Rv | CCGTGTCTCAGTCCCAATCT |  |  |  |  | 37.5% |  |  |
| 2 | Mobil-577F | GCTCGTAGGTGGTTCGTCGC | *Mobiluncus mulieris* | Pre-melt phase at 94 °C for 2 min and denaturation at 94 °C for 30 s; Annealing at 62 °C for 30 s, and extension at 72 °C for 1 min (30 cycles with an additional 5 min of extension step) | 449 bp | *16S* rRNA | 100.0% | N/d | ^116^ |
|  | M.mulie-1026R | CCACACCATCTCTGGCATG |  |  |  |  |  |  |  |
| 3 | Gard154-Fw | CTCTTGGAAACGGGTGGTAA | *Gardnerella* spp. | Pre-melt phase at 94 °C for 2 min and denaturation at 94 °C for 30 s; Annealing at 60 °C for 30 s, and extension at 72 °C for 1 min (30 cycles with an additional 5 min of extension step) | 301 bp | *16S* rRNA | 100.0% | N/d | ^115^ |
|  | Gard154-Rv | TTGCTCCCAATCAAAAGCGGT |  |  |  |  |  |  |  |
| 4 | Primer E1 | ATCAAGTACAGTTAGTCTT | *Enterococcus faecalis* | Pre-melt phase at 94 °C for 5 min and denaturation at 94 °C for 30 s; Annealing at 54 °C for 90 s, and extension at 72 °C for 1 min (30-32 cycles with an additional 5 min of extension step) | 941 bp | *ddl* | 100.0% | Increase of the annealing temperature at 54° C | ^117^ |
|  | Primer E2 | ACGATTCAAAGCTAACTG |  |  |  |  |  |  |  |
| 5 | adk F | ATTCTGCTTGGCGCTCCGGG | *Escherichia coli* | Pre-melt phase at 95 °C for 2 min and denaturation at 95 °C for 1 min; Annealing at 57 °C for 1 min, and extension at 72 °C for 2 min (32 cycles with an additional 5 min of extension step) | 583 bp | *adk* | 49.0% | Increase of the annealing temperature at 57° C; samples validated by API 20E strips | ^118^ |
|  | adk R | CCGTCAACTTTCGCGTATTT |  |  |  |  | 98.0% |  |  |
| 6 | SC1F | CGGAGATTTTCTCAATAAGGACCAC | *Candida albicans* | Pre-melt phase at 94 °C for 3 min and denaturation at 94 °C for 40 s; Annealing at 60 °C for 30 s, and extension at 72 °C for 2 min (32 cycles with an additional 5 min of extension step) | 670 bp | *KER1* | 100.0% | N/d | ^119^ |
|  | SC1R | AGTCAATCTCTGTCTCCCCTTGC |  |  |  |  |  |  |  |

Legend: N/d – non-determined

**Supplementary Table 2**. Parameters used for the diagnosis of vaginal infections.

| **Infection** | **Symptoms** | **Discharge** | **Odor** | **Diagnosis** | **Reference** |
| --- | --- | --- | --- | --- | --- |
| Vulvovaginal Candidiasis | Pruritus | Thick, white to yellow | Absent | Microscopic examination (Gram-stained smears and Wet mount preps included signs of yeast cells and/or pseudo hyphae budding yeasts in high number (average of 5> yeast cells and/or pseudo hyphae per field) in more than two in a total of ten microscopic fields), medical survey (symptoms and vaginal discharge) and growth culture (positive culture in Chocolate and Blood Agar and/or Sabouraud dextrose agar) | ^27,120^ |
| Aerobic Vaginitis | Inflammation | Yellow | Foul, rotten | Microscopic examination (Gram-stained smears and Wet mount preps included signs of the absence or low number of *Lactobacillus* morphotypes (average of <5 cells per field), positive for cocci or coarse bacilli in high number (average of ≥ 20 cells per field), presence of parabasal epithelial cells representing >10% of the epithelial cells, and/ or positive for leukocytes) and medical survey (symptoms and vaginal discharge) | ^70,71^ |
| Bacterial Vaginosis | Irritation, 50% asymptomatic | Thin, white to gray, homogeneous | Fishy | Microscopic examination (Gram-stained smears classified with a total score of 7-10 in Nugent score, indicating signs of >20% clue cells, absence or low number of *Lactobacillus* morphotypes (average of <5 cells per field), and high number of small gram-variable or gram-negative rods and curved gram-negative rods (average of ≥ 20 cells per field) and medical survey (symptoms and vaginal discharge) | ^26,27^ |

**Supplementary Table 3**. Univariable logistic regression analyses of the statistical values between sociodemographic or behavioral variables among women and each type of vaginal microbiota evaluated in this study.

| **Type of vaginal microbiota** | **Sociodemographic or behavioral variables** | ***P*-value** | **OR** | **95% CI** | **Adjusted *P*-value** |
| --- | --- | --- | --- | --- | --- |
| **Healthy Microbiota** | **Age** | | | |  |
|  | 21 – 30 | 0.455 | 1.21 | (0.73 – 2.01) | 0.677 |
|  | 31 – 40 | 0.703 | 1.17 | (0.53 – 2.57) | 0.703 |
|  | 41 – 50 | 0.508 | 1.46 | (0.48 – 4.47) | 0.677 |
|  | Over 50 | 0.087 | 0.32 | (0.09 – 1.18) | 0.348 |
|  | **Education Level** | | | |  |
|  | ≤ Basic | 0.803 | 1.25 | (0.22 – 7.25) | 0.803 |
|  | Secondary | 0.286 | 1.32 | (0.79 – 2.20) | 0.572 |
|  | **Occupation** | | | |  |
|  | Housewife | 0.506 | 0.60 | (0.13 – 2.72) | 0.506 |
|  | Unprofessional | **0.020*** | **0.38** | **(0.17 – 0.86)** | 0.060 |
|  | Professional | 0.487 | 0.83 | (0.48 – 1.41) | 0.506 |
|  | **Civil Status** | | | |  |
|  | Free Union | 0.176 | 0.40 | (0.11 – 1.51) | 0.264 |
|  | Married | 0.325 | 1.39 | (0.72 – 2.66) | 0.325 |
|  | Divorced | 0.176 | 0.40 | (0.11 – 1.51) | 0.264 |
|  | **Sexual Partner** | | | |  |
|  | Having | **0.020*** | **1.64** | **(1.08 – 2.47)** | **0.020*** |
|  | **Contraceptive Use** | | | |  |
|  | Yes | 0.135 | 1.37 | (0.91 – 2.08) | 0.135 |
|  | **Birth Control Methods** | | | |  |
|  | Condom | 0.570 | 1.15 | (0.71 – 1.87) | 0.760 |
|  | Hormonal Contraception | **0.040*** | **2.03** | **(1.03 – 3.99)** | 0.160 |
|  | Combined | 0.218 | 1.53 | (0.78 – 3.01) | 0.436 |
|  | Others | 0.954 | 0.97 | (0.30 – 3.09) | 0.954 |
| **Intermediate Microbiota** | **Age** | | | |  |
|  | 21 – 30 | 0.908 | 1.05 | (0.47 – 2.32) | 0.908 |
|  | 31 – 40 | 0.638 | 0.72 | (0.18 – 2.82) | 0.908 |
|  | 41 – 50 | 0.899 | 1.11 | (0.22 – 5.63) | 0.908 |
|  | Over 50 | 0.427 | 1.98 | (0.37 – 10.60) | 0.908 |
|  | **Education Level** | | | |  |
|  | ≤ Basic | 0.789 | 1.36 | (0.14 – 12.87) | 0.789 |
|  | Secondary | 0.416 | 0.73 | (0.34 – 1.56) | 0.789 |
|  | **Occupation** | | | |  |
|  | Housewife | 0.694 | 1.54 | (0.18 – 13.22) | 0.789 |
|  | Unprofessional | 0.369 | 1.68 | (0.54 – 5.20) | 0.789 |
|  | Professional | 0.789 | 1.12 | (0.49 – 2.55) | 0.789 |
|  | **Civil Status** | | | |  |
|  | Free Union | 0.953 | 1.07 | (0.13 – 8.77) | 0.953 |
|  | Married | 0.813 | 0.89 | (0.33 – 2.38) | 0.953 |
|  | Divorced | 0.953 | 1.07 | (0.13 – 8.77) | 0.953 |
|  | **Sexual Partner** | | | |  |
|  | Having | **0.015*** | **0.45** | **(0.24 – 0.86)** | **0.015*** |
|  | **Contraceptive Use** | | | |  |
|  | Yes | **0.003**** | **0.38** | **(0.20 – 0.72)** | **0.003**** |
|  | **Birth Control Methods** | | | |  |
|  | Condom | **0.008**** | **0.31** | **(0.13 – 0.74)** | **0.032*** |
|  | Hormonal Contraception | **0.021*** | **0.18** | **(0.04 – 0.77)** | **0.042*** |
|  | Combined | 0.404 | 0.67 | (0.26 – 1.72) | 0.539 |
|  | Others | 0.950 | 0.95 | (0.20 – 4.54) | 0.950 |
| **Bacterial Vaginosis** | **Age** | | | |  |
|  | 21 – 30 | 0.958 | 0.97 | (0.34 – 2.78) | 0.999 |
|  | 31 – 40 | 0.683 | 1.36 | (0.31 – 6.00) | 0.999 |
|  | 41 – 50 | 0.992 | 0.99 | (0.11 – 9.00) | 0.999 |
|  | Over 50 | 0.999 | 0.00 | (0.00 – N/d) | 0.999 |
|  | **Education Level** | | | |  |
|  | ≤ Basic | 0.999 | 0.00 | (0.00 – N/d) | 0.999 |
|  | Secondary | 0.829 | 1.13 | (0.37 – 3.42) | 0.999 |
|  | **Occupation** | | | |  |
|  | Housewife | 0.999 | 0.00 | (0.00 – N/d) | 0.999 |
|  | Unprofessional | 0.633 | 0.61 | (0.08 – 4.72) | 0.950 |
|  | Professional | 0.483 | 0.64 | (0.49 – 2.55) | 0.950 |
|  | **Civil Status** | | | |  |
|  | Free Union | 0.999 | 0.00 | (0.00 – N/d) | 0.999 |
|  | Married | 0.546 | 0.63 | (0.14 – 2.79) | 0.819 |
|  | Divorced | 0.517 | 0.52 | (0.24 – 16.94) | 0.819 |
|  | **Sexual Partner** | | | |  |
|  | Having | 0.771 | 0.88 | (0.38 – 2.06) | 0.771 |
|  | **Contraceptive Use** | | | |  |
|  | Yes | 0.382 | 1.50 | (0.60 – 3.73) | 0.382 |
|  | **Birth Control Methods** | | | |  |
|  | Condom | 0.128 | 2.14 | (0.80 – 5.68) | 0.512 |
|  | Hormonal Contraception | 0.725 | 0.75 | (0.15 – 3.72) | 0.863 |
|  | Combined | 0.863 | 0.87 | (0.18 – 4.31) | 0.863 |
|  | Others | 0.581 | 1.85 | (0.21 – 16.26) | 0.863 |
| **Aerobic Vaginitis** | **Age** | | | |  |
|  | 21 – 30 | 0.160 | 0.61 | (0.30 – 1.22) | 0.280 |
|  | 31 – 40 | 0.211 | 0.43 | (0.12 – 1.61) | 0.280 |
|  | 41 – 50 | 0.280 | 0.32 | (0.04 – 2.56) | 0.280 |
|  | Over 50 | **0.026*** | **4.46** | **(1.20 – 16.66)** | 0.104 |
|  | **Education Level** | | | |  |
|  | ≤ Basic | 0.710 | 1.53 | (0.16 – 14.64) | 0.945 |
|  | Secondary | 0.945 | 1.03 | (0.48 – 2.22) | 0.945 |
|  | **Occupation** | | | |  |
|  | Housewife | 0.161 | 3.32 | (0.62 – 17.81) | 0.299 |
|  | Unprofessional | 0.199 | 1.98 | (0.70 – 5.59) | 0.299 |
|  | Professional | 0.728 | 1.15 | (0.52 – 2.52) | 0.728 |
|  | **Civil Status** | | | |  |
|  | Free Union | 0.939 | 0.92 | (0.11 – 7.55) | 0.939 |
|  | Married | 0.595 | 0.77 | (0.29 – 2.04) | 0.893 |
|  | Divorced | 0.364 | 2.11 | (0.42 – 10.48) | 0.893 |
|  | **Sexual Partner** | | | |  |
|  | Having | 0.971 | 0.99 | (0.54 – 1.81) | 0.971 |
|  | **Contraceptive Use** | | | |  |
|  | Yes | 0.957 | 1.02 | (0.55 – 1.88) | 0.957 |
|  | **Birth Control Methods** | | | |  |
|  | Condom | 0.635 | 1.19 | (0.59 – 2.39) | 0.697 |
|  | Hormonal Contraception | 0.690 | 0.82 | (0.31 – 2.16) | 0.697 |
|  | Combined | 0.646 | 0.78 | (0.28 – 2.21) | 0.697 |
|  | Others | 0.697 | 1.37 | (0.28 – 6.65) | 0.697 |
| **Candidiasis** | **Age** | | | |  |
|  | 21 – 30 | 0.866 | 0.87 | (0.17 – 4.55) | 0.999 |
|  | 31 – 40 | 0.998 | 0.00 | (0.00 – N/d) | 0.999 |
|  | 41 – 50 | 0.999 | 0.00 | (0.00 – N/d) | 0.999 |
|  | Over 50 | 0.999 | 0.00 | (0.00 – N/d) | 0.999 |
|  | **Education Level** | | | |  |
|  | ≤ Basic | 0.999 | 0.00 | (0.00 – N/d) | 0.999 |
|  | Secondary | 0.744 | 1.43 | (0.17 – 12.02) | 0.999 |
|  | **Occupation** | | | |  |
|  | Housewife | 0.999 | 0.00 | (0.00 – N/d) | 0.999 |
|  | Unprofessional | 0.429 | 2.42 | (0.27 – 21.49) | 0.999 |
|  | Professional | 0.864 | 0.83 | (0.10 – 7.19) | 0.999 |
|  | **Civil Status** | | | |  |
|  | Free Union | 0.999 | 0.00 | (0.00 – N/d) | 0.999 |
|  | Married | 0.997 | 0.00 | (0.00 – N/d) | 0.999 |
|  | Divorced | 0.999 | 0.00 | (0.00 – N/d) | 0.999 |
|  | **Sexual Partner** | | | |  |
|  | Having | 0.117 | 0.27 | (0.05 – 1.39) | 0.117 |
|  | **Contraceptive Use** | | | |  |
|  | Yes | 0.567 | 1.62 | (0.31 – 8.45) | 0.567 |
|  | **Birth Control Methods** | | | |  |
|  | Condom | 0.266 | 2.65 | (0.48 – 14.68) | 0.999 |
|  | Hormonal Contraception | 0.816 | 1.33 | (0.12 – 14.98) | 0.999 |
|  | Combined | 0.998 | 0.00 | (0.00 – N/d) | 0.999 |
|  | Others | 0.999 | 0.00 | (0.00 – N/d) | 0.999 |
| **Coinfections** | **Age** | | | |  |
|  | 21 – 30 | 0.561 | 1.59 | (0.34 – 7.48) | 0.748 |
|  | 31 – 40 | 0.076 | 4.83 | (0.85 – 27.57) | 0.304 |
|  | 41 – 50 | 0.453 | 2.56 | (0.22 – 29.83) | 0.748 |
|  | Over 50 | 0.999 | 0.00 | (0.00 – N/d) | 0.999 |
|  | **Education Level** | | | |  |
|  | ≤ Basic | 0.999 | 0.00 | (0.00 – N/d) | 0.999 |
|  | Secondary | 0.066 | 0.38 | (0.13 – 1.07) | 0.132 |
|  | **Occupation** | | | |  |
|  | Housewife | 0.999 | 0.00 | (0.00 – N/d) | 0.999 |
|  | Unprofessional | **0.026*** | **4.88** | **(1.21 – 19.63)** | 0.078 |
|  | Professional | 0.089 | 2.71 | (0.86 – 8.53) | 0.134 |
|  | **Civil Status** | | | |  |
|  | Free Union | **0.000***** | **16.65** | **(3.63 – 76.28)** | **0.000***** |
|  | Married | 0.735 | 1.31 | (0.28 – 6.13) | 0.735 |
|  | Divorced | 0.198 | 4.16 | (0.47 – 36.53) | 0.297 |
|  | **Sexual Partner** | | | |  |
|  | Having | 0.792 | 0.87 | (0.32 – 2.39) | 0.792 |
|  | **Contraceptive Use** | | | |  |
|  | Yes | 0.700 | 0.82 | (0.30 – 2.25) | 0.700 |
|  | **Birth Control Methods** | | | |  |
|  | Condom | 0.625 | 0.73 | (0.21 – 2.56) | 0.999 |
|  | Hormonal Contraception | 0.848 | 1.15 | (0.29 – 4.58) | 0.999 |
|  | Combined | 0.863 | 0.87 | (0.18 – 4.31) | 0.999 |
|  | Others | 0.999 | 0.00 | (0.00 – N/d) | 0.999 |

Legend: Univariable logistic regression analyses were conducted to examine how different subcategories (independent variables) were associated with the presence of each type of vaginal microbiota (dependent variables) in each category. The following subcategories of each category were used as reference for statistical analysis: Under 20 in Age; University in Education Level; Student in Occupation; Single in Civil Status; Not Having in Sexual Partner; No in Contraceptive Use; None or Don’t answer in Birth Control Methods. Odds ratios *(OR)* and 95% confidence intervals *(CI)* obtained as measurements of relative risks and the assessments of independent risk factors for vaginal infection establishment. A value of *P* <0.05 and 95% confidence intervals were considered significant for the test: * *P* ≤ 0.05; ** *P* ≤ 0.01; *** *P* ≤ 0.001. N/d – Non-determined. All initial values of *P* <0.05 obtained by univariable logistic regression analyses were then evaluated through Benjamini-Hochberg (BH) adjustment to detect false discovery rate (FDR) for conducting multiple comparisons. These *P*-values evaluated by BH adjustment were illustrated in the table as adjusted *P*-values.
